# LAG3 blockade coordinates with microwave ablation to promote CD8^+^ T cell-mediated anti-tumor immunity

**DOI:** 10.1186/s12967-022-03646-7

**Published:** 2022-09-30

**Authors:** Dong Shao, Yaping Chen, Hao Huang, Yingting Liu, Junjun Chen, Dawei Zhu, Xiao Zheng, Lujun Chen, Jingting Jiang

**Affiliations:** 1 Department of Tumor Biological Treatment, The Third Affiliated Hospital of Soochow University, Changzhou, 213003 Jiangsu Province China; 2 Jiangsu Engineering Research Center for Tumor Immunotherapy, The Third Affiliated Hospital of Soochow University, Changzhou, Jiangsu Province China; 3 Institute of Cell Therapy, The Third Affiliated Hospital of Soochow University, Changzhou, Jiangsu Province China; 4 Department of Gastroenterology, The Third Affiliated Hospital of Soochow University, Changzhou, Jiangsu Province China; 5 State Key Laboratory of Pharmaceutical Biotechnology, Nanjing University, Nanjing, 210023 Jiangsu People’s Republic of China

**Keywords:** Microwave ablation, Immune checkpoint inhibitors, LAG3, Combinational immunotherapy

## Abstract

**Background:**

The immune checkpoint inhibitors (ICIs) combined with other therapeutic strategies have shown exciting results in various malignancies, and ICIs have now become the gold standard for current cancer treatment. In several preclinical and clinical investigations, ablation coupled with immunotherapy has proved to be quite effective. Our previous studies have shown that ablation coupled with ICI is a potential anti-cancer regimen for colorectal cancer liver metastases (CRLM). Furthermore, we have reported that following microwave ablation (MWA), the expression of LAG3 is up-regulated in tumor microenvironment (TME), indicating that LAG3 is implicated in the regulation of immunosuppressive immune response, and combination therapy of MWA and LAG3 blockade can serve as a promising therapeutic strategy against cancer.

**Methods:**

The expression of LAG3 was investigated in this study utilizing a preclinical mouse model treated with MWA. Moreover, we monitored the tumor development and survival in mice to assess the anti-cancer effects of MWA alone or in combination with LAG3 blockade. Flow cytometry was also used to phenotype the tumor-infiltrating lymphocytes (TILs) and CD8^+^ T cell effector molecules. We finally analyzed the single-cell RNA sequencing (scRNA-seq) data of infiltrating CD45^+^ immune cells in the tumors from the MWA alone and MWA combined with LAG3 blockade groups.

**Results:**

After MWA, the expression of LAG3 was up-regulated on sub-populations of TILs, and introducing LAG3 blockade to MWA postponed tumor development and extended survival in the MC38 tumor model. Flow cytometry and scRNA-seq revealed that LAG3 blockade in combination with MWA markedly boosted the proliferation and the function of CD8^+^ TILs, leading to altered myeloid cells in the TME.

**Conclusion:**

Combination therapy of LAG3 blockade and MWA was a unique therapeutic regimen for some solid tumors, and such combination therapy might reprogram the TME to an anti-tumor manner.

**Supplementary Information:**

The online version contains supplementary material available at 10.1186/s12967-022-03646-7.

## Background

As a local minimally invasive treatment, microwave ablation (MWA) has been widely used in the treatment of many solid tumors [1, 2]. MWA can kill cells, which uses electromagnetic waves to generate heat and trigger antigen release and even immune responses [3]. MWA has been shown to offer various advantages over other types of thermal ablation across studies [4]. MWA causes higher volumes of necrosis, higher ablation rates, and increased homogeneity of the decaying region, allowing it to vaporize larger lesions [5, 6]. Several studies have also shown that MWA combined with immune therapy can stimulate a robust anti-tumor immune response and has promising results [3, 7–11]. For example, it has been suggested that in the tumor model established by 4T1 breast cancer cells, the MWA can activate the T-cell immune response, and the combination therapy based on MWA can significantly induce the Th1-type anti-tumor response [10]. Moreover, we have previously confirmed that MWA combined with TIGIT blockade exerts synergistic effects against cancer in contrast to MWA or TIGIT blockade alone [12].

Immune checkpoint inhibitor (ICI)-based immunotherapies have demonstrated broad advantages and durable clinical outcomes in cancer treatments [13–15]. However, only a subset of patients acquires substantive benefits from immunotherapy. This discrepancy suggests that it is urgently necessary to reveal the underlying mechanism. LAG3, also called CD233, is an inhibitory receptor that is highly expressed on activated T cells, natural killer (NK) cells, and plasmacytoid dendritic cells (DCs)[16–18]. LAG3-targeted immunotherapies have been tested as an important anti-tumor agent in lots of clinical trials for multiple types of cancer [19]. It goes either as a single blockade strategy or in combination with other marketed ICIs. For example, the recent clinical trial (NCT03470922) has reported that a combination of LAG-3 and PD-1 blockade can provide a greater benefit with regard to progress-free survival (PFS) than PD-1 blockade alone in patients with previously untreated metastatic or unresectable melanoma [15]. Despite its late stage in clinical trials, the role of LAG3 in the immune cellular network has not yet been fully addressed. Our previous studies have shown that radiofrequency ablation (RFA) combined with PD-1 blockade, or MWA combined with TIGIT blockade, can serve as important combinational therapeutic strategies [9, 12]. Moreover, we have also analyzed publicly available single-cell RNA-sequencing (scRNA-seq) data from the pancreatic ductal adenocarcinoma (PDAC) mouse model with and without RFA therapy, and found that LAG3 expression is up-regulated in CD8^+^ T and CD4^+^ T cell subsets after RFA, suggesting the potential possibilities for the design of combination therapeutic strategy [20].

In the present study, we treated mice with MWA or LAG3 blockade and phenotyped their anti-tumor immune responses in a mouse colon cancer MC38 model. We found that MWA could greatly induce the expression of LAG3 on tumor-infiltrating lymphocytes (TILs) in MC38 tumors. These findings suggested that LAG3 expression, which was up-regulated as an immune self-restrictive molecule after T cell activation, could play a crucial role in combination therapy with MWA. Meanwhile, in the MC38 tumor model, introducing LAG3 blockade to MWA extended survival and postponed tumor development. By attracting CD8^+^ TILs penetrating the tumor microenvironment (TME), LAG3 blockade combined with MWA increased the proliferation and activities of CD8^+^ T cells while reshaping myeloid cells. These findings supported the idea that LAG3 blockade combined with MWA might be a unique treatment regimen that improved anti-tumor immunity synergistically.

## Material and methods

### Cell line and mice

The MC38 cells (mouse colon cancer cell line) in the present study have been used in our previous report [12]. MC38 cells were maintained in DMEM supplemented with 10% (v/v) fetal bovine serum (FBS, Gibco, Thermo Fisher Scientific, USA), 100 U/mL penicillin, and 100 µg/mL streptomycin. Cavens Laboratory Animals provided the 6–8 week-old C57BL/6 mice (male) and kept them in a particular pathogen-free (SPF) facility (Changzhou, China). All animal studies were carried out in accordance with protocols authorized by the Third Affiliated Hospital of Soochow University’s Ethics Committee.

### Animal models, MWA treatment and LAG3 blockade therapy

Each C57BL/6 mouse had a total of 3 × 10^6^ MC38 tumor cells implanted subcutaneously into their bilateral sides (1.5 × 10^6^ MC38 cells for each side) to establish the tumor model according to our previous report [12]. Only after the tumor volume reached roughly 300 mm^3^ was MWA can be performed on the tumor on the right flank. MWA was conducted using an ablation electrode (Microwave Ablation Antennas, Canyon Medical Inc., Jiangsu Nanjing) percutaneously inserted in the center of the tumor as reported in our previous study [12]. The treatments lasted 2–4 min at 70 °C and 8 W. LAG3 blockade was then intraperitoneally administered four times every 3 days, starting on day 1 after MWA. An anti-LAG3 mAb (Clone C9B7W, BioXcell, USA) was administered at a dose of 200 μg per mouse per injection. The diameters of the tumors on the left flank were measured every other day, and the tumor volume was calculated using the formula as follows: Volume = Length × Width^2^/2. Tumor growth curve and the overall survival (OS) were observed and charted.

### Flow cytometry

Tumor tissues from the left tumors were collected and digested with Liberase TL (Catalog No. 05401020001, Roche) and DNase I (Catalog No. 10104159001, Roche) for 30 min at 37 °C, and prepared into single-cell suspension as reported in our previous study [12]. Antibodies used in staining and flow analysis included CD45 (Clone 30-F11), CD3 (Clone 17A2), CD4 (Clone GK1.5), CD8 (Clone 53–6.7), NK1.1 (Clone PK136), LAG3 (Clone C9B7W), Foxp3 (Clone 3G3), CD11b-BV650 (Clone M1/70), MHC-II (Clone AF6-120.1), CD11c (Clone N418), CD206 (Clone C068C2), F4/80 (Clone BM8) and CD103 (Clone M290). Intracellular cytokine staining was also performed as described in previous study [12]. Following stimulation, the cells were labeled with antibodies against surface markers, fixed, and permeabilized using the Invitrogen Fixing/Permeabilization Solution kit’s manufacturer’s instructions. Antibodies against TNF-α (Clone MP6-XT22) and IFN-γ (Clone XMG1.2) were used to stain the fixed cells. A BD FACS Aria II flow cytometer was used to collect data, which were then analyzed using FlowJo software.

### scRNA-Seq

The procedure described above was used to make single-cell suspensions of tumor cells from the left tumors. The cells were enriched for FACS sorting using the CD45 (TIL) Microbead Mouse Kit (Catalog No. 130-110-618, Miltenyi Biotec, Leiden, the Netherlands) and labeled with the antibodies Ghost DyeTM Violet 510 Viability Dye (Cell Signaling Technology) and Percp-Cy5.5-CD45 (Clone 30-F11). A BD Aria II device was used to sort about 5 × 10^5^ CD45^+^ cells per sample. Single cells were sorted into flow tubes based on the FACS analysis, and cell viability was determined by calculating the AOPI to guarantee adequate cell quality. The cell suspension was then put onto the chromium single-cell controller (10X Genomics) to form single-cell gel beads in the emulsion according to the manufacturer’s directions, with 300–600 viable cells per microliter as measured by CountStar. An S1000TM Touch Thermal Cycler (Bio-Rad) was used to perform single-cell transcriptome amplification at 53 °C for 45 min, followed by incubation at 85 °C for 5 min and storage at 4 °C. The quality of the cDNA templates was tested using Agilent 4200 equipment after they were produced and amplified (performed by CapitalBio Technology, Beijing).

### scRNA-Seq data processing, integration of multiple scRNA-Seq data, dimension reduction, and unsupervised clustering

The Cell Ranger Single-Cell Software Suite was used to match the freshly generated scRNA-seq data acquired from 10X Genomics to the mm10 mouse reference genome and quantify it. The pre-filtered data obtained by Cell Ranger was used to construct a Seurat object with the R package Seurat (version 3.2.3). With the DoubletFinder package, doublets were eliminated. The overall UMI count, the number of identified genes, and the fraction of mitochondrial gene count per cell were all used to apply quality control to cells in a stepwise way. Cells with more than 5,000 UMI counts and 10% mitochondrial gene counts were specifically screened out. The workflow in Seurat was used to analyze single-cell data for dimension reduction and unsupervised clustering analysis. Using the Find Variable Features function with the option “n features = 2,000,” 2,000 highly variable genes were chosen for downstream analysis. The data were then integrated and a new matrix with 3,000 features was created, in which the possible batch effect was regressed, using the Integrated Data function. To minimize the dimensionality of the scRNA-seq dataset, principal component analysis (PCA) was conducted on an integrated data matrix. The top 40 PCs were submitted to downstream analysis using Seurat’s Elbow plot program. The primary cell clusters were found using Seurat’s Find Clusters tool with a resolution of 0.1. The clusters were then displayed using two-dimensional tSNE or UMAP plots. Each cell was classified into a recognized biological cell type using conventional markers established in a previous work.

### Differential gene expression analysis

DEGs were identified between clusters using the EdgeR package (version 3.28.1). The calcNormFactors function was used to normalize the raw data from the Seurat object using TMM (trimmed mean of M-values), and the estimateDisp function was used to estimate the dispersion of gene expression levels. The DEGs were chosen for display using the Seurat package’s DotPlot function.

### Trajectory analysis

Single-cell trajectories were built with the Monocle2 R package (version 2.14.0) that introduced pseudotime. Genes were filtered by the following criteria: expressed in more than 10 cells. Then, the ECDF plot was performed by the ggplot2 package geom_ecdf() function to compare different states between two samples.

### Statistical analysis

Statistical analysis was performed using Graphpad Prism v8. The log-rank test was used for comparisons in overall survival. Two-way ANOVA was used for comparing tumor growth curves. The two-tailed un-paired Student’s *t*-test was used to compare two groups and the ANOVA test was used for multiple comparisons.

## Results

### LAG3 is up-regulated in TILs upon MWA

**Fig. 1:**
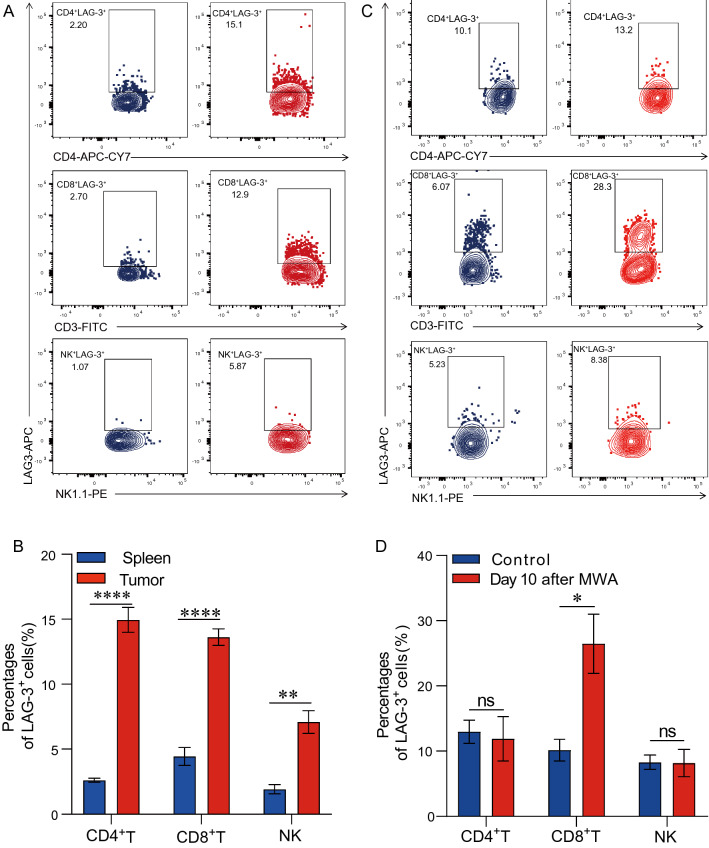
LAG3 expression is up-regulated upon MWA. **A** and **B**. Representative flow cytometry plot and a corresponding quantitative plot showing the proportion of LAG3-expressing cells in tumor or spleen, indicating that LAG3 was highly expressed on CD4^+^T cells, CD8^+^T cells and NK cells in MC38 tumor tissues (n = 4). **C** and **D**. Representative flow cytometry plot and corresponding quantitative plot showing the increased proportion of LAG3^+^CD8^+^T cells in MC38 tumors on day 10 after MWA treatment (n = 4). ^*^P < 0.05, ^**^P < 0.01, ^****^P < 0.0001

**Fig. 2:**
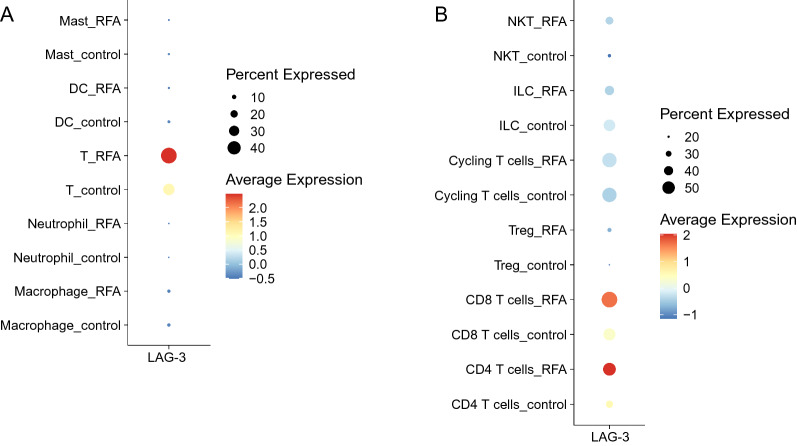
LAG3 expression is up-regulated after RFA. **A** and **B**. Dot plot shows the expression of LAG3 in major cell populations in the published RFA single-cell dataset [20]

As a co-inhibitory receptor, LAG3 is highly expressed in hyperactivated T cells [21]. Using the MC38 tumor model, we first investigated the LAG3 expression on TILs. Our data revealed that the LAG3 expression was much higher on TILs, including CD4^+^ TILs, CD8^+^ TILs, and NK cells, compared with splenocyte compartments (Fig. 1A and B). Next, we assessed the LAG3 expression in the distant tumor environment after MWA treatment in the MC38 tumor model established as in our previous work [12]. The expression of LAG3 was significantly enhanced in CD4^+^ TILs, CD8^+^ TILs, and NK cells on day 10 after MWA (Fig. 1C and D). We then analyzed publicly available scRNA-seq data from the PDAC mouse model with and without RFA therapy [20]. Consistently, we found that LAG3 expression was also up-regulated in different T cell populations, especially in CD8^+^ T and CD4^+^ T cell subsets after RFA (Fig. 2A and B).

### MWA and LAG3 blockade synergistically delay tumor growth

**Fig. 3:**
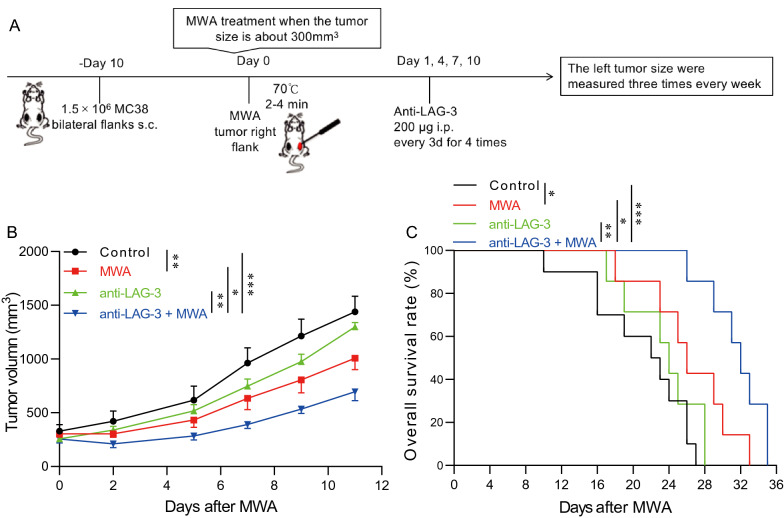
Anti-LAG3 synergizes with MWA to inhibit tumor outgrowth **A**. Schematic drawing for the therapeutic treatment in our present study. A total of 3 × 10^6^ MC38 cells were subcutaneously inoculated into the bilateral flanks (1.5 × 10^6^ MC38 cells for each side) of C57BL/6 mice. Mice bearing the MC38 tumor were divided into four groups. **B**. The tumor size on the left flank was measured three times every week after MWA, showing that the LAG3 blockade plus MWA significantly decreased the tumor growth (n = 5 for each group). **C**. Log-rank survival analysis shows LAG3 blockade plus MWA treatment significantly prolonged the OS of the tumor-bearing mice (n = 10 for the control group, and for the other three groups, n = 7 respectively). ^*^P < 0.05, ^**^P < 0.01, ^***^P < 0.001

MWA induced a robust immune response and up-regulated the LAG3 expression on T cells. Subsequently, we sought to combine MWA and LAG3 blockade to assess whether MWA would benefit the therapeutic efficacy of LAG3 blockade, following the protocol we have used in our previous study (Fig. 3A) [12]. MC38 tumor-bearing mice were divided into four groups. The control group was given the isotype control, the MWA group was given MWA and the isotype control, the LAG3 blockade group was given the anti-LAG3 monoclonal antibody, and the MWA plus LAG3 blockade group was given MWA and the anti-LAG3 monoclonal antibody. MC38 tumors were unaffected by LAG3 blockade alone, whereas tumors in either the MWA group or MWA combined with LAG3 blockade group showed significantly delayed tumor outgrowth (Fig. 3B). Furthermore, the OS of the MWA group and MWA combined with the LAG3 blockade group was significantly longer compared with the control group and LAG3 blockade alone group (Fig. 3C). These results indicated that the MWA plus LAG3 blockade could promote the anti-tumor immune response by targeting LAG3-expressing TILs in the TME.

### scRNA-seq analysis reveals a transcriptional landscape in TME

**Fig. 4:**
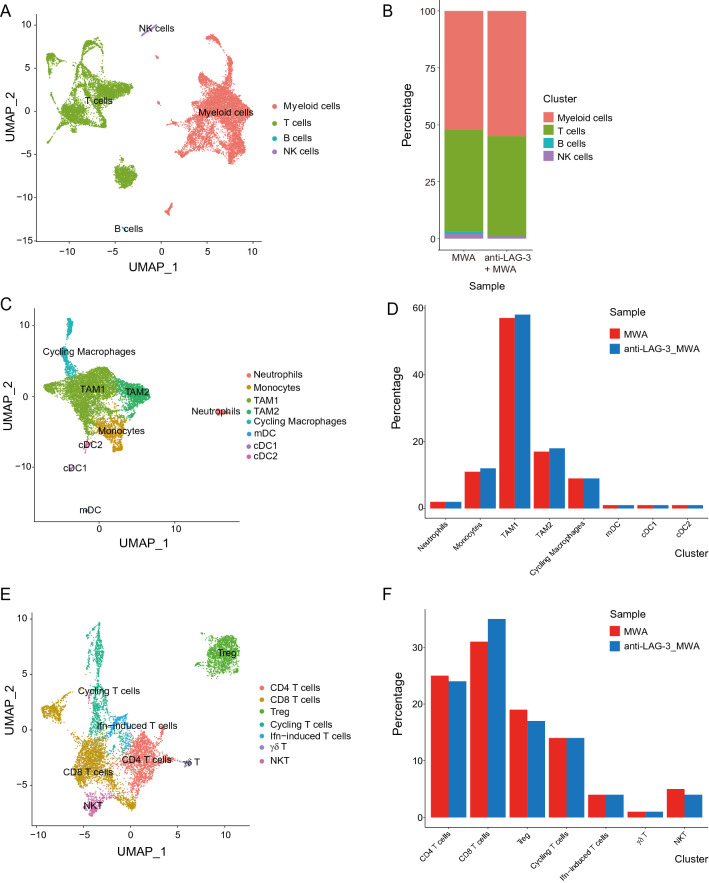
Transcriptional landscape of TME in the MWA alone and MWA combined with LAG3 blockade groups. **A** and **B**. UMAP analysis of all the cells in TME of the MWA alone or MWA combined with the LAG3 blockade group and the proportions of different subpopulations in each sample (MWA group: 8971 cells; MWA combined with the LAG3 blockade group: 8733 cells). **C** and **D**. UMAP analysis of all the myeloid cells in TME of the MWA alone or MWA combined with LAG3 blockade group and the proportions of different subpopulations in each sample. **E** and **F**. UMAP analysis of all the lymphocytes in TME of the MWA alone or MWA combined with LAG3 blockade group and the proportions of different subpopulations in each sample

To have a deeper understanding of what was happening in the TME, we performed scRNA-seq experiments using the transplant tumor model MC38. We sorted the tumor-infiltrating CD45^+^ immune cells. Based on the gene expression, all the CD45^+^ immune cells could be divided into four clusters, namely the myeloid cells, T cells, B cells, and NK cells (Fig. 4A). Furthermore, we looked into the proportion of each cluster between the MWA group and the combination group, and found that the proportion of myeloid cells was increased a little bit in the combination group (Fig. 4B). Subsequently, we conducted the subcluster analysis of the myeloid cells and T cells. We found that the proportions of monocytes, TAM1 and TAM2 cells were slightly increased in the combination group (Fig. 4C and D). Besides, among the T cell clusters, we could only find slight changes of the percentages of CD4^+^ T cells, Treg cells, and NKT cells bwtween the two groups. We found that the proportion of CD8^+^ T cells was increased in the MWA combined with the LAG3 blockade group based on scRNA-seq and flow analysis (Fig. 4E and F). Even so, we also carried out the flow cytometry analysis to confirm the differences of sub-populations in MWA and MWA combined with the LAG3 blockade groups, and we found that the percentage of Treg cells was not significantly changed (Additional file 1: Figure S1A and S1B). We also did not find significant changes of DCs, Macrophages, or even DC1, DC2, type I macrophages and type II macrophages in MWA and MWA combined with the LAG3 blockade groups (Additional file 1: Figure S1C, S1D and S1E).

### Combination therapy of MWA and LAG3 blockade results in increased TILs

**Fig. 5:**
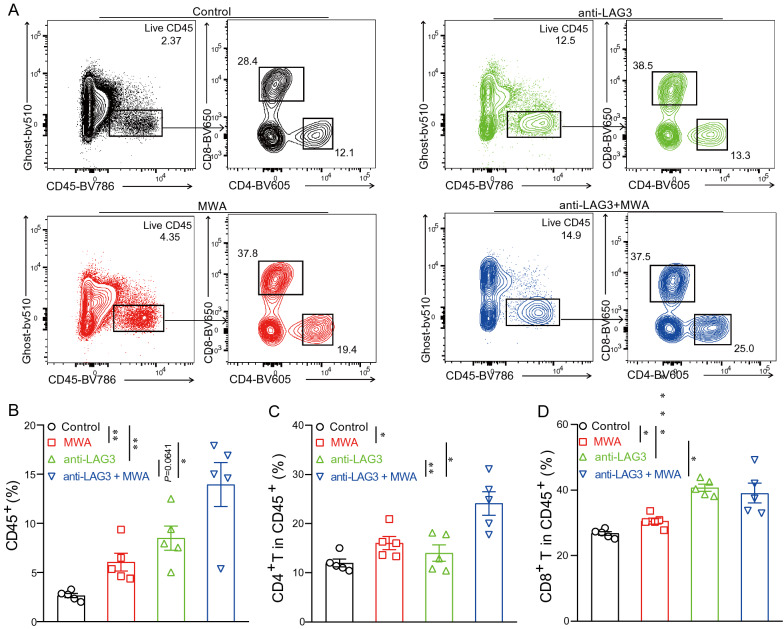
Anti-LAG3 synergizes with MWA to induce immune cell infiltration. **A**. Representative flow cytometry plots showing the proportions of live CD45^+^ TILs, CD4^+^ TILs, and CD8^+^ TILs. **B**. Frequencies of CD45^+^ TILs in different treatment groups. **C**. Frequencies of CD4^+^ TILs in different treatment groups. **D**. Frequencies of CD8^+^ TILs in different treatment groups. (5 mice for each group). ^*^P < 0.05, ^**^P < 0.01, ^****^P < 0.0001

In order to better understand the underlying mechanisms that were responsible for enhanced anti-tumor activities in the MWA combined with the LAG3 blockade group, we checked the proportions of TILs in the tumors from different groups (Fig. 5). Compared with tumors harvested from control mice, the proportion of CD45^+^ TILs was increased by 3–4 folds in tumors isolated from the combination group (Fig. 5A and B). Within the CD45^+^ TILs, we reported that the proportions of CD4^+^ T and CD8^+^ T cells were significantly higher compared with the control group, MWA group alone, and LAG3 blockade alone group (Fig. 5C and D). Overall, these results showed that MWA combined with LAG3 blockade resulted in more inflamed TME.

### Combination therapy of MWA and LAG3 blockade alters CD8^+^ TILs

**Fig. 6:**
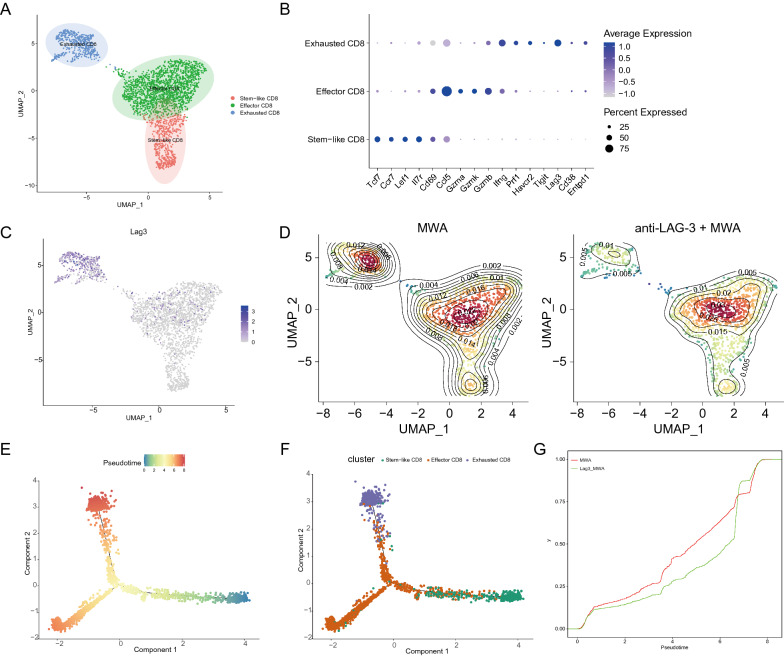
Single-cell analysis of CD8^+^ TILs. **A**. UMAP analysis of all the CD8^+^ TILs. **B**. Dot plot showing the expression of specific genes among three clusters. **C**. Feature plot showing the distribution of LAG3 expression in UMAP space. **D**. Density plot showing the different distribution of three clusters between two treatment conditions. **E**. Trajectory analysis of all the CD8^+^ TILs colored by the pseudo time. **F**. Trajectory analysis of all the CD8^+^ TILs colored by different CD8^+^ TIL clusters. **G**. ECDF plot exhibit the diference of pseudotime regarding to LAG3 blockade and combination therapy groups

In order to have a better understanding of how MWA combined with LAG3 blockade altered the CD8^+^ TILs, we did a detailed sub-clustering of all the CD8^+^ T cells from the MWA group and MWA combined with the LAG3 blockade group. We identified three different clusters, which were stem-like CD8, effector CD8, and exhausted CD8 clusters (Fig. 6A). Figure 6B shows the DEGs in each cluster. The stem-like CD8 cluster had high expressions of naïve genes, such as *Tcf7*, *Ccr7*, *Lef1*, and *Il7r*. In contrast, T cell function genes, such as *Gzma*, *Gzmk*, and *Gzmb*, were highly expressed in the effector CD8 cluster. For the exhausted CD8^+^ T cells cluster, we found that multiple immune checkpoint molecules and *Ifng* were significantly expressed in such cluster (Fig. 6B). LAG3 was exclusively expressed in the exhausted CD8^+^ T cell cluster, and the proportion of this cluster was significantly decreased after the combination treatment (Fig. 6C and D). We next performed trajectory analyses and found that CD8^+^ T cells underwent extensive differentiation from stem-like CD8^+^ T cells to effector CD8^+^ T cells and exhausted CD8^+^ T cells cluster (Fig. 6E and F). Then, we also applied the ECDF plot to exhibit the cumulative distribution of pseudotime regarding to LAG3 blockade and combination therapy groups, and further demonstrated that the pseudotime of combination therapy group was lower than that of LAG3 blockade group, suggesting the higher percentage of effector CD8^+^ T cells in combination therapy group (Fig. 6G).

### MWA and LAG3 blockade synergistically enhance CD8^+^ T cell functions

**Fig. 7:**
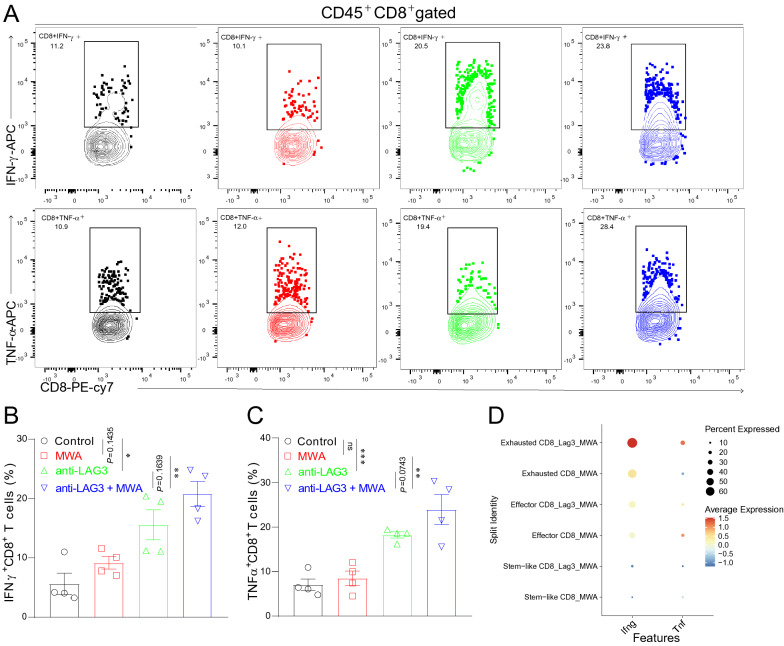
Anti-LAG3 coordinates with MWA to promote CD8^+^ T cell functions **A**–**C**. Representative flow cytometry plots and corresponding quantitative bars showing the proportion of IFN-γ and TNF-α expressing CD8^+^ TILs in different treatment groups. **D**. Dot plot showing the gene expression of *Ifng* and *Tnf* in scRNA-seq dataset. ^*^P < 0.05, ^**^P < 0.01, ^***^P < 0.001

We next found that the production of IFN-γ and TNF-α in CD8^+^ T cells in the MWA group was similar to that in the control group (Fig. 7A–C). Also, we found that IFN-γ and TNF-α were higher in CD8^+^ T cells from the LAG3 blockade group or combination group compared with the control group or MWA group (Fig. 7A–C). Next, we also examined the expressions of *Ifng* and *Tnf* genes in scRNA-seq dataset. Consistently, the average gene expressions of *Ifng* and *Tnf* were up-regulated in the combination group compared with the MWA alone group (Fig. 7D). These results showed that LAG3 blockade enhanced MWA-induced anti-tumor immunity by increasing the proportion of functional CD8^+^ T cells.

### Combination therapy of MWA and LAG3 blockade alters the cell–cell communication across all immune subsets

**Fig. 8:**
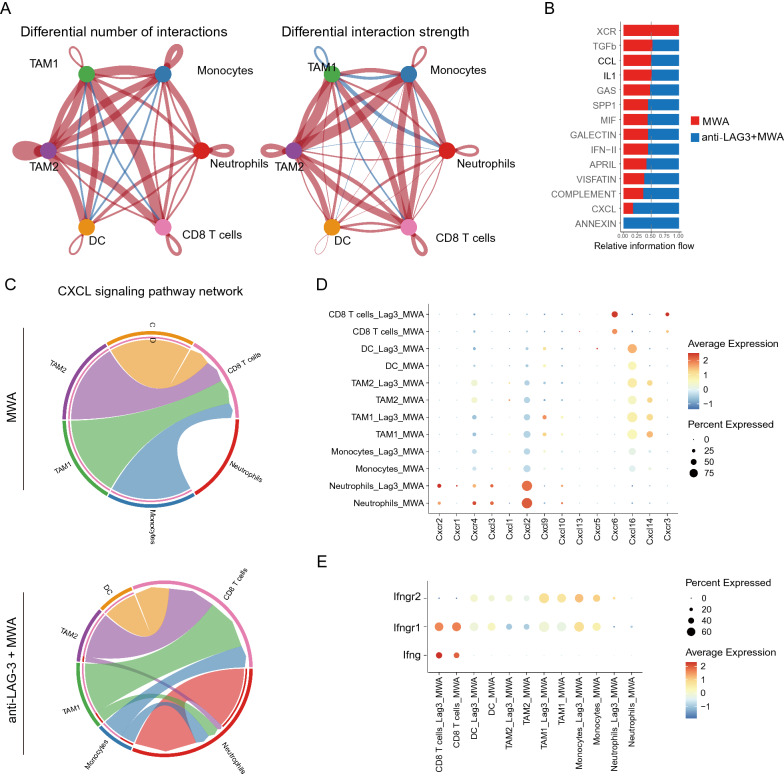
Combined therapy of MWA and LAG3 blockade alters the cell–cell communication across all immune subsets. **A**. The cell–cell communication among immune cell populations between the MWA group and MWA in combination with LAG3 blockade group. **B**. Bar plot showing the relative information flow between the MWA group and MWA combined with the LAG3 blockade group. **C**. Chord plot showing the specific cell–cell interaction with the CXCL signaling pathway. **D**. Dot plot showing the genes of the CXCL signaling pathway among different clusters. **E**. Dot plot showing the genes of IFNG signaling pathway among different clusters

To fully examine cell–cell communication among different immune cell sub-populations, we used Cell Chat to quantitatively characterize and compare the predicted intercellular communication networks across the MWA and combination therapy groups as described in our previous study [22]. In the combination group, the interactions between myeloid cells and CD8^+^ T cells were significantly enhanced both in the number of interactions and the strength (Fig. 8A), indicating a more immune-activated TME. We next assessed the specific receptor-ligand pairs and found that the IFN and CXCL pathways were more enriched in the combination therapy group compared with the MWA alone group (Fig. 8B). Besides, we investigated the CXCL signaling pathway network, and from the chord plot, we could clearly see more interactions in the combination therapy group compared with the MWA alone group (Fig. 8C). On the other hand, we examined the specific genes in the CXCL signaling pathway (Fig. 8D) and IFN signaling pathway (Fig. 8E) in the MWA alone group and combination therapy group. We found that the expressions of these genes were up-regulated in the combination therapy group, which was consistent with the findings that the interactions among the CXCL pathway and IFN pathway were stronger in the combination therapy group.

## Discussion

A great deal of success has been achieved in the treatment of various malignancies with combination immunotherapy based on ICI [23, 24]. In many preclinical and clinical settings, ablation coupled with ICIs has produced unprecedented OS benefits for cancer patients. Shi et al. demonstrated that RFA could induce robust T cell-mediated anti-tumor immune responses in distant tumors, and Chen et al. have also shown that combination therapy of MWA and TIGIT blockade synergistically promotes anti-tumor immune response in mouse colon cancer model [9, 12]. Furthermore, RFA does not prevent tumor recurrence in certain individuals, indicating that alternative immune-suppression pathways were involved in the TME after FRA [25]. Many clinical trials comparing RFA and MWA have demonstrated that both two methods represented similar efficacy and safety, while MWA has technical advantages in terms of reduced heat sink effect and faster ablation [4, 26]. In the present study, we found that MWA can greatly induce the expression of LAG3 on TILs, especially on CD8^+^ TILs, indicating that the combination regimen could provide benefits. Therefore, it’s of great imporatance to reveal the therapeutic effect of combinational strategy against cancer and explore its possible clinical application prospect.

As we know, LAG3 is highly expressed on activated T cells, NK cells, and plasmacytoid DCs [16–18]. Similar to the CD4 molecule, LAG3 has four distinct Ig-like domains, sharing the same ligand as CD4, the MHC-II [27]. LAG3 blockade results in enhanced T cell expansion and up-regulated function signature in vitro [28, 29]. Upon tumorigenesis and chronic viral infection, such as the LCMV C13 strain, LAG3 is up-regulated in T cells along with other immune inhibitory receptors, such as PD-1, TIM3, and TIGIT [30–32]. Moreover, currently, as the next generation of immune checkpoint therapy in cancer, LAG-3 has been confirmed as an important candidate target, for example, the clinical trials NCT00732082, NCT00349934, NCT02614833, NCT01968109 and NCT02460224, have shown that LAG-3 blockade can not only improve the antitumor immune responses but also can potentiate other forms of immunotherapy [33]. However, the molecular mechanism of how LAG3 affects the T cell function in the scenario of cancer still remains to be illustrated. It has been suggested that the antagonistic mAb against LAG3 could blockade the interaction between LAG3 and MCH-II molecules expressed by tumor and/or immune cells, leading to the promotion of tumor cell apoptosis [34]. Another phase I clinical trial has reported that, IMP321, a soluble form of LAG3, can have an objective response rate (ORR) of 50% in metastatic breast cancer when combined with paclitaxel [35]. Therefore, we aimed to identify whether LAG3 blockade could significantly enhance the MWA-induced anti-tumor immune response by introducing more inflamed tumors and more functional CD8^+^ T cells.

In summary, MWA dramatically induced the expression of LAG3 on different TILs sub-populatios in TME, and anti-LAG3 treatment in combination with MWA, could significantly suppress tumor development, increase effector CD8^+^ TILs, and restore the tumor-killing function of exhausted CD8^+^ T cells. The present similar mechanism was also found in our previous studies, such as RFA combined with PD-1 blockade, and MWA combined with TIGIT blockade, it is necessary to evaluated the therapeutic efficacy of the above three combined treatment methods separately, or even perform the investigation of triple therapy strategy, such as MWA plus PD-1 and LAG3 blockade [9, 12]. In fact, we tried MWA plus TIGIT and PD-1 blockade, and the results revealed that the triple therapy showed significant advantages in contrast to MWA plus TIGIT, MWA plus PD-1, or even MWA and TIGIT alone (data not shown). Furthermore, the LAG3 blockade plus MWA dramatically improved T cell interaction, demonstrating that the combination of LAG3 blockade and MWA could effectively suppress the inhibitory signals on T cells in a synergistic manner. Therefore, LAG3 blockade combined with MWA might be employed in the clinical setting to reprogram the TME in an anti-cancer manner, revealing the potential value of the clinical application.

## Conclusions

Our present study first reported that the combination of LAG3 blockade and MWA could extend the survival and postponed tumor development in the MC38 bilateral tumor model, and the combination therapy could reprogram the TME to an anti-tumor manner via promoting the functions of CD8^+^TILs.

## Supplementary Information

**Additional file 1: Figure S1. **Flow cytometry analysis was used to confirm the differences of sub-populations in MWA and MWA combined with the LAG3 blockade groups. **A**. Gating strategy of tumor infiltrating Foxp3^+^Tregs. **B**. The percentage of Tregs was not significantly changed in MWA and MWA combined with the LAG3 blockade groups (n=5 for each group). **C**. Gating strategy of sub-sets of tumor infiltrating DCs and macrophages. **D**. There were not significant changes of DCs, DC1, DC2 in MWA and MWA combined with the LAG3 blockade groups (n=5 for each group). **E**. There were not significant changes of Macrophages, type I macrophages and type II macrophages in MWA and MWA combined with the LAG3 blockade groups (n=5 for each group).

## Data Availability

All data generated or analyzed during this study are included in this published article.
