# Supplementary figures and images for: LAG3 blockade coordinates with microwave ablation to promote CD8+ T cell-mediated anti-tumor immunity
Source: J Transl Med. 2022 Sep 30;20:433. doi: 10.1186/s12967-022-03646-7 (PMC9524118; doi:10.1186/s12967-022-03646-7)

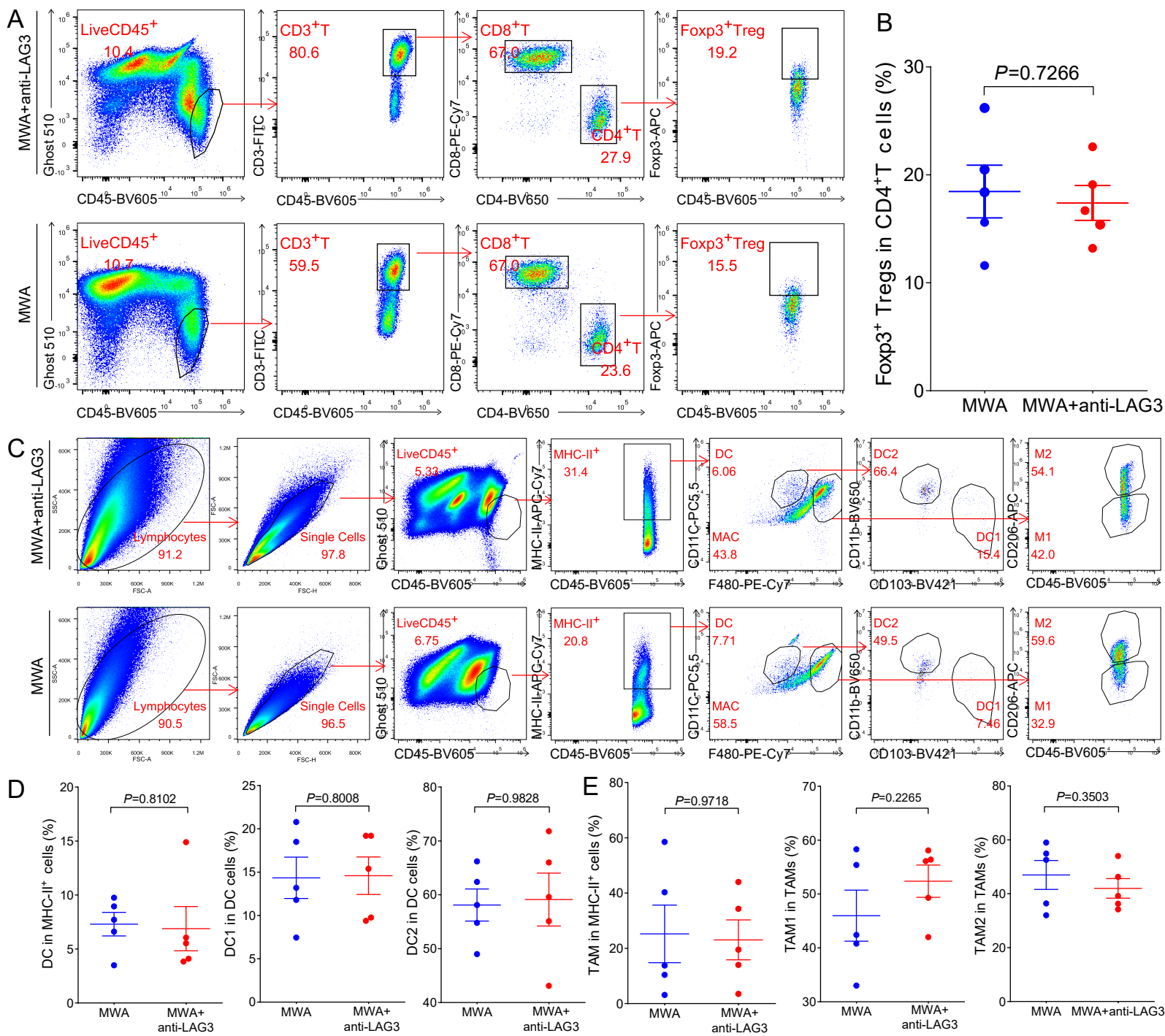

Supplement: Supplementary file 1 — Additional file 1: Figure S1. Flow cytometry analysis was used to confirm the differences of sub-populations in MWA and MWA combined with the LAG3 blockade groups. A. Gating strategy of tumor infiltrating Foxp3+Tregs. B. The percentage of Tregs was not significantly changed in MWA and MWA combined with the LAG3 blockade groups (n=5 for each group). C. Gating strategy of sub-sets of tumor infiltrating DCs and macrophages. D. There were not significant changes of DCs, DC1, DC2 in MWA and MWA combined with the LAG3 blockade groups (n=5 for each group). E. There were not significant changes of Macrophages, type I macrophages and type II macrophages in MWA and MWA combined with the LAG3 blockade groups (n=5 for each group). [file 12967_2022_3646_MOESM1_ESM.pdf]
